# Trend of incompleteness of the Robson Classification variables in the Live Birth Information (SINASC) in the state of Paraná, Brazil, 2014-2020

**DOI:** 10.1590/S2237-96222024V33E2023632.EN

**Published:** 2024-02-02

**Authors:** Larissa Pereira Falavina, Elizabeth Fujimori, Maicon Henrique Lentsck

**Affiliations:** 1 Universidade de São Paulo, Programa de Pós-Graduação em Enfermagem, São Paulo, SP, Brazil; 2 Universidade Estadual do Centro-Oeste do Paraná, Departamento de Enfermagem, Guarapuava, PR, Brazil

**Keywords:** Cesarean Section, Health Information Systems, Descriptive Epidemiology, Vital Statistics, Cesárea, Sistemas de Información en Salud, Epidemiología Descriptiva, Estadísticas Vitales, Cesárea, Sistemas de Informações em Saúde, Epidemiologia Descritiva, Estatísticas Vitais

## Abstract

**Objective:**

To assess the incompleteness of the Robson Classification variables in the Live Birth Information System (*Sistema de Informação sobre Nascidos Vivos* - SINASC), in the state of Paraná, and its trend, 2014-2020.

**Methods:**

This was a time-series study that analyzed six variables, according to health macro-regions. Incompleteness was classified (percentage of “ignored” and “blank fields”) as follows: excellent (< 1.0%); good (1.0-2.9%); regular (3.0-6.9%); poor (≥ 7.0%). Prais-Winsten regression was used to estimate trends.

**Results:**

A total of 1,089,116 births were evaluated. The variable “cesarean section before the onset of labor” was classified as poor in 2014 (39.4%) and 2015 (44.3%) in the state and in all macro-regions, but with a decreasing trend in incompleteness. The variables “gestational age” in the North and Northwest macro-regions, and “parity” and “number of fetuses” in the Northwest macro-region showed an increasing trend.

**Conclusion:**

Most of the variables evaluated showed low percentages of incompleteness with a decreasing trend, but there is a need to improve the completion of some variables.

## Study contributions

**Main results:**

The majority of variables showed a low percentage of incompleteness with a decreasing trend. It is essential to enhance the data completeness for the variables "cesarean section before the onset of labor" and "induced labor".

**Implications for services:**

The low percentage of incompleteness in SINASC data contributes to the good quality of the Robson Classification, which can be used to reduce the high rates of caesarean sections.

**Perspectives:**

Proposal of strategies to ensure good data quality, especially for variables with 'regular' and 'poor' completeness, involving training for data completion and professional qualification for the use of the Robson Classification.

## RESUMEN

**Objetivo:**

Evaluar la incompletitud de las variables de la Clasificación de Robson en el Sistema de Información de Nacidos Vivos (SINASC) de Paraná y su tendencia, 2014-2020.

**Métodos:**

Estudio de series temporales que analizó seis variables, según macrorregiones de salud. La incompletitud (percentaje de campos “ignorados” y “en blanco”) se clasificó como: excelente (< 1,0%); buena (1,0-2,9%); regular (3,0-6,9%); mala (≥ 7,0%). Se utilizó la regresión de Prais-Winsten para estimar tendencia.

**Resultados:**

Se evaluaron 1.089.116 nacimientos. “Cesárea antes del inicio del trabajo de parto” se clasificó como mala en 2014 (39,4%) y 2015 (44,3%) en Paraná y en todas las macrorregiones, pero con tendencia decreciente de incompletitud. Las variables “edad gestacional”, “paridad” y “número de fetos” mostraron tendencia creciente.

**Conclusión:**

La mayoría de las variables evaluadas mostraron porcentajes bajos de incompletitud, con una tendencia decreciente, pero es necesario mejorar el cumplimiento de algunas variables que mostraron una tendencia creciente de incompletitud.

## RESUMO

**Objetivo:**

*Avaliar a incompletude das variáveis da Classificação de Robson no Sistema de Informação sobre Nascidos Vivos (Sinasc) do Paraná e sua tendência, 2014-2020.*

**Métodos:**

*Estudo de séries temporais que analisou seis variáveis, segundo macrorregionais de saúde. Classificou-se a incompletude (percentual de campos “ignorados” e “em branco”) em: excelente (< 1,0%); bom (1,0-2,9%); regular (3,0-6,9%); ruim (≥ 7,0%). Utilizou-se regressão de Prais-Winsten para estimar tendências.*

**Resultados:**

*Foram avaliados 1.089.116 nascimentos. A variável “cesárea antes do trabalho de parto iniciar” classificou-se como ruim, em 2014 (39,4%) e 2015 (44,3%), no estado, e em todas as macrorregionais, porém com tendência decrescente de incompletude. As variáveis “idade gestacional” no Norte e Noroeste, e “paridade” e “número de fetos” no Noroeste apresentaram tendência crescente.*

**Conclusão:**

*A maioria das variáveis avaliadas mostrou baixos percentuais de incompletude com tendência decrescente, mas é preciso melhorar o preenchimento de algumas variáveis.*

## INTRODUCTION

Robson Classification^1^ is a tool that classifies pregnant women into groups with a lower/higher chance of undergoing a cesarean section.^2^ ^)-(^ ^3^ In 2015, the World Health Organization (WHO) proposed its use in all childbirth facilities.^4^ ^)^ High cesarean section rates pose a significant health problem, adversely effecting maternal and child health.^5^

Since 1990, Brazil has employed the Live Birth Information System (*Sistema de Informação sobre Nascidos Vivos* - SINASC), which collects data from the Live Birth Certificate (LBC). As of 2009, new fields were included in the LBC and, consequently, in the SINASC,^6^ ^)-(^ ^7^ highlighting the number of weeks of gestation, fetal presentation, and labor induction, automatically generating the Robson Classification.

Studies on the quality of SINASC have shown good completion.^7^ ^)-(^ ^11^ Studies conducted in the state of Paraná, between 1996 and 2018, found good information quality,^9^ ^)-(^ ^12^ however, not all variables necessary to generate the Robson Classification were assessed, such as the onset of labor and fetal presentation. Paraná state has one of the highest cesarean section rates in the country (62.6%), while the state of Roraima has one of the lowest rates (35.2%).^13^ ^),(^ ^14^ In 2020, Paraná passed Law No. 20.127,^15^ granting women the right to choose the type of delivery, which could impact these statistics.

The objective of this study was to assess the incompleteness of the Robson Classification variables in the SINASC, in the state of Paraná, and its trend from 2014 to 2020.

## METHODS

This was a time series study that analyzed seven variables from SINASC in Paraná, a state in southern Brazil with an estimated population of 11,597,484 inhabitants in 2021, which has one of the highest Human Development Index scores and it is one of the largest economies in the country.^16^ It is organized into four Health Macro-regions (East, West, North and Northwest).^16^ SINASC database, from 2014 to 2020, was used, available at the www.datasus.gov.br. Data extraction was performed using the TabWin tool (available on the website) on March 3, 2021.

The Robson Classification, based on five characteristics, classifies pregnant women into ten groups: groups 1 to 4 - with a lower likelihood of cesarean section; group 5 - with some likelihood; and groups 6 to 10 - with a higher likelihood.^2^ ^),(^ ^3^

In order to obtain the five obstetric characteristics that comprise Robson’s Classification^1^, it was necessary to assess the following variables: “onset of labor”, which takes into account two variables from LBC/SINASC: “was labor induced?” and “did cesarean section occur before the onset of labor?”; “fetal presentation”; “parity”, related to the variables “number of previous vaginal deliveries” and “number of previous cesarean sections” from LBC/SINASC; “gestational age” and “number of fetuses”. The names of each variable mentioned above are described in Box 1. 

Incompleteness, assessed by the percentage of “ignored” and “blank fields”, was classified using criteria employed in another study in Paraná,^9^ which considered excellent (incompleteness < 1.0%); good (1%-2.9%); regular (3%-6.9%); and poor (≥ 7.0%).

The trend of incompleteness was estimated using Prais-Winsten regression, which corrects for residual temporal autocorrelation. The trend was interpreted as follows: stationary (p-value > 0.05); decreasing [p-value < 0.05 and negative regression coefficient (β1)] or increasing [p-value < 0.05 and positive regression coefficient (β1)]. The analyses were performed according to health macro-regions, using the Stata software, version 13. Although using publicly available secondary data, this study was approved by the Research Ethics Committee of the Escola de Enfermagem da Universidade de São Paulo (CAAE 58854522.1.0000.5392).

**Box 1:** Characteristics used to compose the Robson Classification, their definitions and designations in the Live Birth Information System (Sistema de Informações sobre Nascidos Vivos - SINASC) database

Characteristics used in the Robson Classification	Definition	Variable designations in SINASC
Onset of labor^a^	Spontaneous, induced or cesarean section before the onset of labor.	STTRABPART = Was labor induced? STCESPARTO = did cesarean section occur before the onset of labor?
Fetal presentation	Position of the fetus in the womb: cephalic, breech or transverse.	TPAPRESENT = Type of fetal presentation.
Parity	Nulliparous: has never given birth. Multiparous: has given birth at least once.	QTDPARTNOR = Number of previous vaginal deliveries. QTDPARTCES = Number of previous caesarean sections.
Gestational age	Corresponds to the gestational age at birth.	SEMAGESTAC = Number of weeks of gestation.
Number of fetuses	Corresponds to the number of fetuses in that pregnancy: single, twins, triplets or more.	GRAVIDEZ = Type of pregnancy.

a) In order to obtain this variable, it was necessary to analyze two variables separately (“Was labor induced?” and “Did cesarean section occur before the onset of labor?”), as they cannot be combined, which justifies the analysis of six variables.

## RESULTS

Between 2014 and 2020, a total of 1,089,116 births were registered on SINASC, in the state of Paraná. The highest percentages of poor incompleteness classification were observed in the variable “cesarean section before the onset of labor”, especially in 2014 (39.4%) and 2015 (44.3%), both in the state and in all health macro-regions, with emphasis on the East and West health macro-regions, where poor or regular classification of incompleteness was observed for the variable “induced labor” until 2017. Other variables showed excellent and good percentages of incompleteness in all macro-regions (Table 1).

The variable “cesarean section before the onset of labor” showed a decreasing trend in incompleteness in all macro-regions (annual changes: -25.5% to -42.0%). The variable “induced labor” presented a decreasing trend in incompleteness in the East and West macro-regions. The variables “parity”, “gestational age” and “number of fetuses” showed an increasing trend in incompleteness in the Northwest macro-region and “gestational age” in the North macro-region (Table 2).

**Table 1:** Percentage of incompleteness of variables from the Live Birth Information System (SINAN) used to compose the Robson Classification, according to health macro-regions, state of Paraná, Brazil, 2014-2020

Macro-regions/Variables	2014	2015	2016	2017	2018	2019	2020
East							
Cesarean section before the onset of labor.	47.6	53.6	8.2	8.5	5.9	7.2	10.0
Induced labor	11.8	11.7	7.1	8.3	6.7	5.8	6.9
Fetal presentation	3.0	2.3	1.4	1.3	0.6	0.6	1.0
Parity	0.8	0.6	0.8	0.2	0.3	0.3	0.5
Gestational age	1.5	1.2	1.0	1.0	0.5	0.6	0.8
Number of fetuses	0.1	0.1	0.2	0.1	0.1	0.1	0.1
West							
Cesarean section before the onset of labor.	34.7	37.2	2.0	2.4	3.1	2.0	1.6
Induced labor	4.2	3.9	4.0	4.0	3.3	3.8	3.5
Fetal presentation	3.2	3.6	3.1	1.4	0.2	0.4	0.3
Parity	0.1	0.2	0.1	0.7	0.1	0.2	0.2
Gestational age	0.5	0.6	1.7	0.6	0.3	0.4	0.5
Number of fetuses	0.1	0.1	0.1	0.1	0.1	-	0.1
North							
Cesarean section before the onset of labor	31.4	34.3	2.8	3.1	2.8	3.8	4.6
Induced labor	1.1	1.4	1.6	3.1	2.4	1.4	1.4
Fetal presentation	1.1	1.2	1.2	1.5	0.9	0.6	0.7
Parity	2.7	3.5	3.4	1.1	0.8	0.4	0.4
Gestational age	0.4	1.2	1.0	1.2	1.1	1.3	1.3
Number of fetuses	0.1	0.1	0.1	0.1	0.1	0.1	0.1
Northwest							
Cesarean section before the onset of labor	24.9	32.8	4.7	2.3	1.5	1.8	1.4
Induced labor	0.2	0.7	0.6	0.9	0.5	0.5	0.6
Fetal presentation	0.6	0.8	0.7	0.3	0.5	0.4	0.4
Parity	0.1	0.1	-	0.1	2.7	0.3	1.1
Gestational age	0.2	0.4	0.3	0.4	0.4	0.5	0.5
Number of fetuses	-	0.1	0.1	0.1	0.1	0.1	0.1
Paraná state							
Cesarean section before the onset of labor	39.4	44.3	5.6	5.5	4.2	4.7	6.1
Induced labor	7.0	6.9	4.6	5.4	4.4	3.8	4.3
Fetal presentation	2.3	2.1	1.6	1.2	0.6	0.5	0.7
Parity	0.9	0.9	1.0	0.4	0.7	0.3	0.5
Gestational age	0.9	0.9	1.0	0.9	0.5	0.7	0.8
Number of fetuses	0.1	0.1	0.1	0.1	0.1	0.1	0.1

a) Classification: excellent (< 1.0%); good (1.0% to 2.9%); regular (3.0% to 6.9%); poor (≥ 7.0%).

**Table 2:** Trend of incompleteness of the variables from SINASC used for the Robson Classification, average annual percentage change and confidence interval according to health macro-regions, state of Paraná, Brazil, 2014-2020

Variables	Annual change^a^	_95%_ **CI** ^b^	Trend
Cesarean section before the onset of labor			
East	-27.5	-44.3;-5.7	Decreasing
West	-41,7	-56.0;-22.7	Decreasing
North	-25.5	-42.8;-2.9	Decreasing
Northwest	-42.0	-55.5;-24.5	Decreasing
Paraná state	-32,3	-48.8;-10.8	Decreasing
Induced labor			
East	-10.0	-13.6;-6.3	Decreasing
West	-2.3	-3.1;-1.5	Decreasing
North	-10.4	-14.0;44.7	Stable
Northwest	2.3	-7.7;13.5	Stable
Paraná state	-6.0	-6.7;-5.2	Decreasing
Fetal presentation			
East	-19.0	-26.8;-10.4	Decreasing
West	-35.0	-45.7;-22.1	Decreasing
North	-1.3	-19.8;21.5	Satable
Northwest	-10.4	-14.3;-6.3	Decreasing
Paraná state	-20.4	-26.6;-13.7	Decreasing
Parity			
East	-13.5	-22.3;-3.8	Decreasing
West	5.3	-15.4;31.1	Stable
North	-24.8	-40.6;-4.9	Decreasing
Northwest	63.0	7.7;146.7	Increasing
Paraná state	-8,8	-19.5;3.2	Stable
Gestational age			
East	-1.9	-20.2;1.9	Decreasing
West	-5.9	-30.2;26.8	Stable
North	12.9	3.8;22.8	Increasing
Northwest	11.4	8.9;13.9	Increasing
Paraná state	-0.1	-5.9;6.0	Stable
Number of fetuses			
East	2.8	-10.7;18.3	Stable
West	-5,4	-10.1;-0.5	Decreasing
North	8.0	-1.2;18.2	Stable
Northwest	3.1	1.7;4.5	Increasing
Paraná state	2.1	-4.9;9.6	Stable

a) Average annual percentage change of incompleteness rates of variables calculated from the β1 of the Prais-Winsten generalized linear regression model. b) 95%CI: 95% confidence interval.

## DISCUSSION

In an unprecedented approach, the quality of data completion for variables comprising the Robson Classification in the SINASC system in Paraná state was assessed. Among the key findings, the variable “cesarean section before the onset of labor” stood out with poor incompleteness, but a decreasing trend, indicating improvement in data completion. “Induced labor” showed poor and regular classification in the East and West macro-regions, respectively, but with a decreasing trend in incompleteness. It is noteworthy that poor incompleteness was related to the highest percentage of information that was not filled in, left blank or ignored.^9^ ^)-(^ ^11^ Three variables showed an increasing trend in incompleteness in two health macro-regions (Northwest and North).

The poor incompleteness of the variable “cesarean section before the onset of labor” in all macro-regions in 2014 and 2015 could be partially justified by the fact that these were the first years of assessing this variable, which was included in SINASC in 2011. However, a national analysis found regular completion (70-90%) of this variable in 2015, but good completion (90-95%) of other variables included in 2011,^7^ indicating the need for periodic training to improve data completion in LBC and SINASC.

The persistence of higher percentages of incompleteness in the East and West macro-regions throughout the study period may be linked to regional disparities within the state, such as the low prenatal care coverage in regions with lower socioeconomic status, found in some health macro-regions of the state. This reveals that economic factors impact health investment and professional training.^17^ ^),(^ ^18^

In order to enhance the generation of the Robson Classification in the SINASC system, aiming to contribute to the reduction of cesarean section rates, it is essential to improve the filling in of the variables “cesarean section before the onset of labor” and “induced labor”, which are related to the characteristic “onset of labor”. It is worth highlighting that only one study evaluating the data completion quality of these variables was identified in the literature, and found similar results.^7^ Therefore, further studies on data completion quality of these variables should be conducted.

Most of the variables analyzed showed very low percentage of incompleteness, corroborating the results of a national study that assessed SINASC data for over 3 million births that occurred in 2002, where Paraná state presented low percentages.^8^ In fact, a study that evaluated the quality of other variables on the SINASC, in Paraná, over a 22-year period (1996 to 2018), showed very low incompleteness percentage in the state,^12^ recommending the use of this information system.^19^ ^),(^ ^20^ In addition, the law passed in Paraná in 2020,^15^ may have contributed to an increase in cesarean section rates in the state, leading to reflection on crucial aspects, such as legislation and political decisions that strongly influence health indicators.

The results obtained represent an advancement compared to previous studies conducted in the state of Paraná,^8^ ^),(^ ^9^ ^),(^ ^12^ between 1996 and 2018, as they analyzed all the variables necessary for the automatic generation of the Robson Classification in SINASC. Despite being one of the information systems with the most satisfactory quality in the country,^8^ ^)-(^ ^11^ ^),(^ ^21^ ^)-(^ ^23^ ^)^ analyses stratified by health macro-regions in the state revealed significant differences, emphasizing the need for training and periodic evaluations related to this topic.

In 2009, the completion of “gestational age” variable was changed to completed weeks of gestation. According to a literature review that analyzed studies published from 2010 to 2018, this variable shows the highest percentages of incompleteness.^21^ In Mato Grosso, there was an increasing trend of incompleteness for this variable in 2011-2012,^10^ as observed in the North and Northwest macro-regions of Paraná, a result also observed in the Northeast region of Brazil, even with the use of a different scale.^24^

The “number of fetuses” showed an incompleteness percentage below 1% in all the years studied, similar to findings in Recife.^25^ However, the increasing trend in incompleteness for this variable, as well as for the variable “parity” identified in this study, indicates the need for attention to these variables.

One limitation of this study is the use of secondary data, subject to the reliability of information filled in by professionals, which may include errors and difficulties in data completion. Despite this, the study of the variables that generate the Robson Classification in the SINASC system and its analysis is fundamental, as it enables the identification of the groups of pregnant women most likely to undergo cesarean section, contributing to the implementation of strategies aimed at reducing cesarean section rates.^2^ ^),(^ ^26^

Thus, the evaluation of the incompleteness of the Robson Classification variables in the SINASC system in the state of Paraná and its trend stands out as a strong point, as it showed that the majority of variables presented low percentages of incompleteness and a decreasing trend, although, improvement in the completion of the “parity”, “gestational age” and “number of fetuses” variables, is still necessary. These variables showed an increasing trend especially in the Northwest macro-region. This diagnosis can support the implementation of public policies by directing strategies for the continuous improvement of SINASC, through periodic analyses and training for those involved. It is worth highlighting that in addition to having an information system with adequate completion, success in reducing cesarean sections requires political will, along with the implementation of comprehensive measures, legislation and public policies.
